# Comparison of characteristics and outcomes of young‐onset versus average onset pancreatico‐biliary adenocarcinoma

**DOI:** 10.1002/cam4.5418

**Published:** 2023-01-09

**Authors:** Thejus Jayakrishnan, Kanika G. Nair, Suneel D. Kamath, Wei Wei, Bassam N. Estfan, Smitha S. Krishnamurthi, Alok A. Khorana

**Affiliations:** ^1^ Taussig Cancer Institute Cleveland Clinic Ohio Cleveland USA; ^2^ Department of Quantitative Health Sciences Cleveland Clinic Ohio Cleveland USA

**Keywords:** biliary cancers, cholangiocarcinoma, disparity, epidemiology, pancreatic adenocarcinoma, young‐onset cancers

## Abstract

**Background:**

Young‐onset gastrointestinal malignancies appear to be increasing in incidence. There are limited data on young‐onset pancreaticobiliary adenocarcinoma (YO‐PBA).

**Methods:**

The study comprised patients with PBA (pancreatic adenocarcinoma, intra‐, and extra‐hepatic cholangiocarcinoma) and included in the National Cancer Database (NCDB) between 2004 and 2017. YO‐PBA was defined as a diagnosis at age less than 50 years. Logistic regression to assess factors associated with YO‐PBA status, and cox proportional hazards modeling to associate relevant factors with overall survival was performed.

**Results:**

The study cohort comprised 360,764 patients, with 20,822 (5.8%) YO‐PBA. YO‐PBA was associated with (*p*‐values<0.0001 for all): male sex (6.3% YO‐male out of all male patients vs. 5.2% YO‐female, OR 1.29, 95% CI 1.25–1.33), Black race (7.9% YO‐Black vs. 5.0% YO‐White, OR 1.72, 95% CI 1.64–1.80), lower income (6.4% YO‐lowest household income based group vs. 5.5% highest, OR 1.08, 95% CI 1.03–1.13). YO‐PBA were more likely to present with stage‐IV disease (6.4% YO‐Stage IV of all stage IV vs. 5.4% YO‐Stage I–III, OR 1.25, 95% CI 1.21–1.29 *p*‐value < 0.0001). Factors associated with overall survival (OS) in non‐operable patients included—sex ‐ male vs. female, HR 1.12 (95% CI 1.08–1.15); race ‐ Black vs. White, HR 1.23 (95% CI 1.06–1.42); income group ‐ lowest vs. highest, HR 1.33 (95% CI 1.27–1.39), and treatment center type ‐ academic vs. nonacademic center, HR 0.87 (95% CI 0.85–0.90).

**Conclusions:**

Socioeconomic factors significantly impact incidence and outcomes for young‐onset pancreaticobiliary adenocarcinoma (YO‐PBA). More work is needed to help understand the mechanisms involved while addressing the disparities.

## Graphical abstract

The incidence and outcomes for young‐onset pancreaticobiliary adenocarcinoma is significantly influenced by socioeconomic factors. Further research is needed to help understand the mechanisms involved while addressing the disparities.

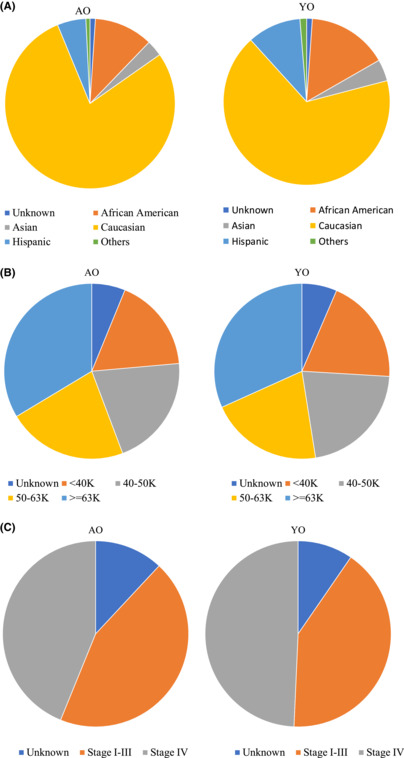

## 1 INTRODUCTION

Pancreatico‐biliary cancers remain among the most aggressive types of cancer. Pancreatic cancer is currently ranked the third leading cause of cancer‐related deaths in the United States with a median 5‐ year overall survival (OS) of approximately 10%.^1^ While outcomes are slightly better for cholangiocarcinoma (5‐year OS 20%), it is still associated with a high mortality rate. Alarming data show an annual average 0.5% rise in age‐adjusted rates for new diagnoses and an annual 0.2% rise in age‐adjusted death rates from pancreatic cancer.^2^ For cholangiocarcinoma, the age‐adjusted death rates have been rising on average by 1.3% each year from 2010 to 2019.^3^

While the median age for diagnosis of pancreatico‐biliary cancers is around 70, approximately 5%–15% are young‐onset diseases defined variably as <40 or <50 years of age at diagnosis, with an increasing incidence.^1^ Patients with young‐onset cancers have several unique challenges compared to patients with average onset disease in terms of cancer diagnosis and access to treatment. Most studies on young‐onset gastrointestinal cancers have focused on colorectal cancer and there have been limited data on young onset pancreatico‐biliary adenocarcinoma (YO‐PBA). It is unclear whether disease characteristics (risk factors, response to treatment) and factors impacting outcomes are comparable to those with a typical age of onset of the disease. The current study aimed to evaluate the characteristics of YO‐PBA and analyze factors associated with outcomes.

## 2 METHODS

### 2.1 Study design and participants

A retrospective cohort analysis using de‐identified data accessed from the National Cancer Database (NCDB) was conducted. The study was exempt from Institutional Review Board (IRB) oversight and did not require Ethics approval. The NCDB was queried for patients diagnosed between 2004 and 2017 with pancreatic adenocarcinoma, intra and extra hepatic cholangiocarcinoma (Disease sites: liver; intrahepatic bile duct; other biliary; pancreas; ICD codes: C221, C240, C241, C248, C249, C250, C251, C252, C253, C254, C257, C258, C259; Histology codes 8140, 8141, 8160, 8180, 8255, 8260, 8261, 8310, 8323, 8440). Patients with all American Joint Commission for Cancer (AJCC) stages were chosen for the study.

### 2.2 Variables

Race/ethnicity was categorized into five categories—Non‐Hispanic White, Non‐Hispanic Black, Asian, Hispanics, and others. These categories were generated based on the following variables available in NCDB—race (identifying the primary race of an individual) and the variable for identifying Spanish/Hispanic origin for a person. Other sociodemographic variables studied were biologic sex available in the dataset (male and female), educational status represented in terms of quartiles of the percentage of persons with less than high school education, and median household income—both of these were according to the residents’ census tract. Assignment of locations were based on data provided by the United States Department of Agriculture Economic Research Service and categorized as rural, urban or metropolitan. Insurance status is captured in the NCDB as it appears on the admission face sheet for the patient and was recoded as insured (Private, Medicaid, Medicare, others) or uninsured. Facility type is assigned in NCDB according to the Commission on Cancer accreditation category. Comorbidity was captured using the Charlson/Deyo comorbidity index.^4^

### 2.3 Definitions and statistical analyses

Young‐onset was defined as age of diagnosis less than 50 years and average onset was defined as age of diagnosis greater than 50 years.

Summary statistics are provided in frequencies and percentages for categorical variables and by median, interquartile range (IQR), and range for data that is quantitiative.^5^ Univariate and multivariate logistic regression was used to assess factors associated with YO‐PBA status and results are represented as odds‐ratio (OR) along with 95% CIs. A variable selection was not performed given the large sample size of NCDB, and all variables used for NCDB studies were included in the analysis.

**FIGURE 1:**
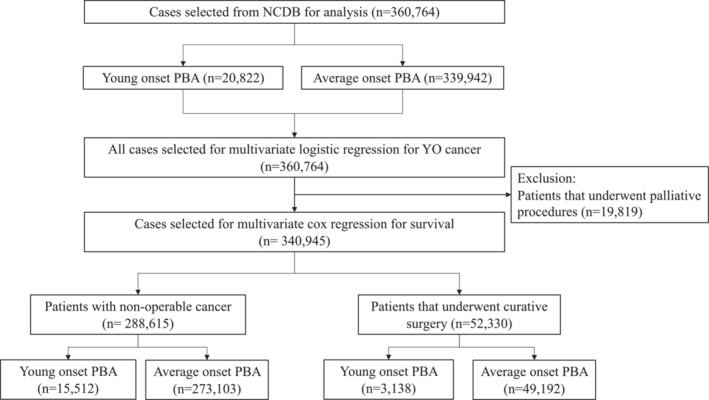
Flow diagram demonstrating selection of patients for the study

Overall survival (OS) was measured in months (m) from day of diagnosis to day of death or censoring (last follow‐up). OS was estimated by Kaplan–Meier method and compared between groups by log‐rank test. Multivariate Cox proportional hazards modeling was performed to associate relevant demographic and socioeconomic factors with overall survival. Given the significant role of curative surgery in improving outcomes for these patients, the group that underwent curative surgery was analyzed separately from those that were non‐operable (patients who underwent palliative surgeries were excluded). The interaction terms between patient characteristics and YO‐PBA status were significant, therefore, YO‐PBA and AO‐PBA were analyzed separately as outlined in Figure 1. All tests were two‐sided and *p*‐values of 0.05 or less were considered statistically significant. Statistical analysis was carried out using SAS Studio 3.7 (SAS Institute, Inc) and R version 4.1 (R Foundation).

## 3 RESULTS

### 3.1 Study population

The baseline characteristics of the cohorts (YO‐PBA and AO‐PBA) are summarized in Table 1. Of 360,764 patients analyzed, 20,822 (5.8%) were YO‐PBA. The number of YO‐PBA increased from 1,288 in 2004 to 1,717 in 2017, a relative percentage increase of 33.3% in comparison to 111.8% increase for AO‐PBA (16,183–34,268). The median age at diagnosis for YO‐PBA was 45 years (IQR 42–48) versus 70 (62–78) years for AO‐PBA. The selection process for the analysis is outlined in Figure 1.

**TABLE 1:** Baseline characteristics of patients selected for the analysis—*n* (%) for categorical variables and median (interquartile range—IQR) for continuous variables

Characteristic	YO‐PBA (*n* = 20822)	AO‐PBA (*n* = 339942)	All patients (*n* = 360764)
Age in years	45 (42‐48)	70 (62‐78)	69 (60‐77)
Tumor type			
Intrahepatic	2742 (13.2)	27361 (8.1)	30103 (8.3)
Extrahepatic	3343 (16.1)	50333 (14.8)	53676 (14.9)
Pancreatic	14737 (70.8)	262248 (77.1)	276985 (76.8)
Sex			
Male	11793 (56.6)	174398 (51.3)	186191 (51.6)
Female	9029 (43.4)	165544 (48.7)	174573 (48.4)
Race			
Non‐Hispanic White	14028 (67.4)	266864 (78.5)	41261 (77.9)
Non‐Hispanic Black	3253 (15.6)	38008 (11.2)	11308 (11.4)
Asian	874 (4.2)	10434 (3.1)	20674 (3.1)
Hispanic	2168 (10.4)	18506 (5.4)	2929 (5.7)
Others	270 (1.3)	2659 (0.8)	41261 (0.8)
Unknown	229 (1.1)	3471 (1.0)	3700 (1.0)
Primary payor			
Government	5223 (25.1)	226352 (66.6)	231575 (64.2)
Private	13125 (63.0)	98035 (28.8)	111160 (30.8)
Not insured	1860 (8.9)	8616 (2.5)	10476 (2.9)
Unknown	614 (3.0)	6939 (2.0)	7553 (2.1)
Median income			
<40K	4058 (19.5)	59179 (17.4)	63237 (17.5)
40–50K	4488 (21.6)	70285 (20.7)	74773 (20.7)
50–63K	4323 (20.8)	75193 (22.1)	79516 (22.0)
≥63K	6608 (31.7)	114271 (33.6)	120879 (33.5)
Unknown	1345 (6.5)	21014 (6.2)	22359 (6.2)
Education (% without high school education in the community)			
≥17.6%	4899 (23.5)	66234 (19.5)	71133 (19.7)
10.9%–17.5%	5341 (25.7)	82232 (24.2)	87573 (24.3)
6.3%–10.8%	5113 (24.6)	91060 (26.8)	96173 (26.7)
<6.3%	4164 (20.0)	80045 (23.6)	84209 (23.3)
Unknown	1305 (6.3)	20371 (6.0)	21676 (6.0)
Comorbidity score			
0	16725 (80.3)	223281 (65.7)	240006 (66.5)
≥1	4097 (19.7)	116661 (34.3)	120758 (33.4)
Location			
Metropolitan	16908 (81.2)	276634 (81.4)	293542 (81.4)
Urban	325 (13.4)	6217 (13.6)	6542 (13.6)
Rural	2791 (1.6)	46179 (1.8)	48970 (1.8)
Unknown	798 (13.4)	10912 (3.2)	11710 (3.3)
Facility type			
Academic/Research program	8550 (41.1)	142145 (41.8)	150695 (41.8)
Non‐Academic	12272 (58.9)	197797 (58.2)	210069 (58.2)
Stage			
Stage I–III	8560 (41.1)	150245 (44.2)	158805 (44.0)
Stage IV	10264 (49.3)	149078 (43.9)	159342 (44.2)
Unknown	1998 (9.6)	40619 (12.0)	42617 (11.8)
Surgery			
Unknown	92 (0.44)	1532 (0.45)	1624 (0.45)
No	15512 (74.5)	273103 (80.3)	288615 (80.0)
Yes	5218 (25.1)	65307 (19.2)	70525 (19.6)
Radiation therapy			
Unknown	423 (2.0)	6987 (2.1)	7410 (2.1)
No	15617 (75.0)	276452 (81.3)	292069 (81.0)
Yes	4782 (23.0)	56503 (16.6)	61285 (17.0)
Chemotherapy			
Unknown	781 (3.8)	13414 (4.0)	14195 (3.9)
No	5268 (25.3)	147893 (43.5)	153161 (42.5)
Yes	14773 (71.0)	178635 (52.3)	193408 (53.6)

### 3.2 Characteristics associated with YO‐PBA

**FIGURE 2:**
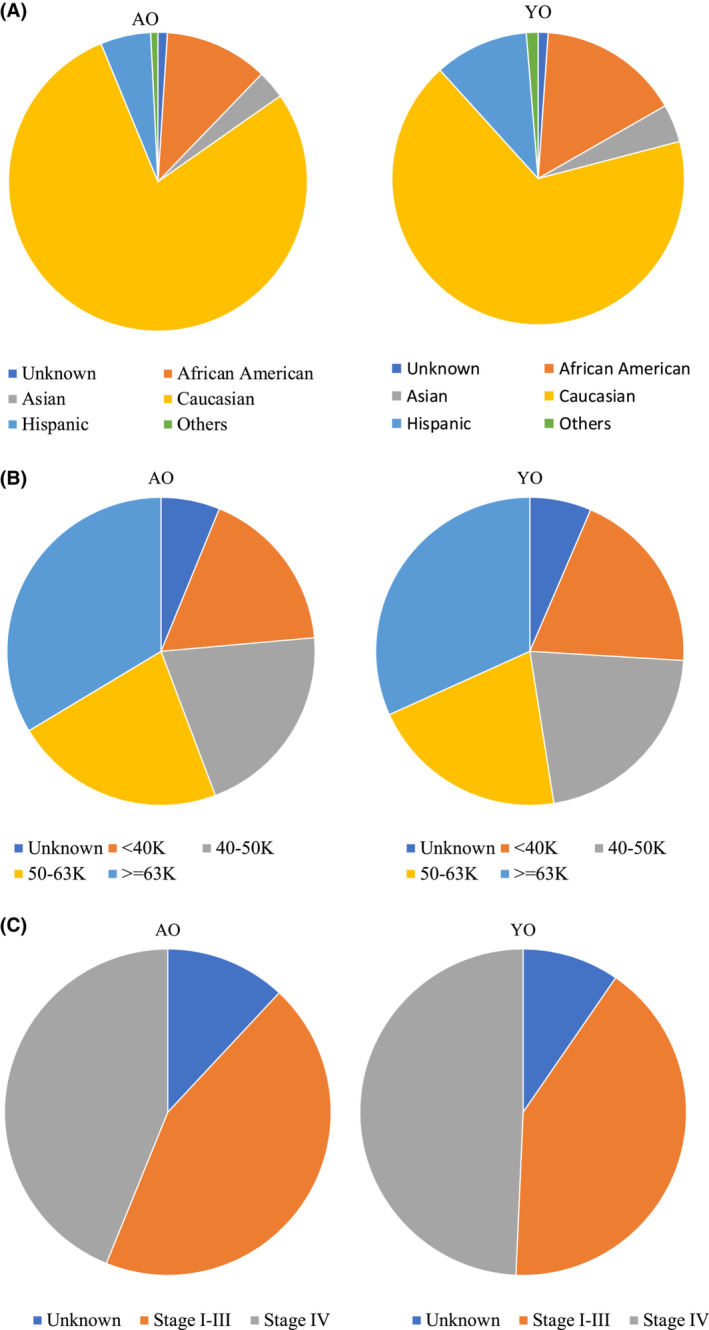
Graphical illustration of the distribution of (A) Race/ethnicity, (B) Income and (C) Stage groups in YO‐PBA and AO‐PBA

On univariate logistic regression analysis, the significant factors associated with YO‐PBA included: sex (56.6% YO‐male vs. 51.3% AO‐male, OR 1.24, 95% CI 1.21–1.28), race (15.6% YO‐non‐Hispanic Black vs. 11.2% AO‐non‐Hispanic Black, OR 1.52, 95% CI 1.46–1.58), income group (19.5% YO‐lowest household income based group vs. 17.4% AO‐lowest household income based group, OR 1.18, 95% CI 1.14–1.23), with a *p*‐values < 0.0001 for all. The variables were incorporated into a multivariable logistic regression model as are outlined in Table 2. On multivariate logistic regression, the statistically significant factors associated with YO‐BPA included: male sex (OR 1.29, 95% CI 1.25–1.34, *p*‐value < 0.0001), other versus non‐Hispanic White (Asian vs. non‐Hispanic White, OR 1.59, 95% CI 1.47–1.72; non‐Hispanic Black vs. non‐Hispanic White, OR 1.72, 95% CI 1.64–1.80, and Hispanics vs. non‐Hispanic White, OR 2.25, 95% CI 2.13–2.38, all *p*‐values < 0.0001), income group (lowest income based group vs. highest, OR 1.08, 95% CI 1.03–1.13, *p*‐value = 0.0025). YO‐PBA were more likely to present with Stage‐IV disease (6.4% of all Stage IV cases were YO‐PBA) versus stage I–III disease (5.4% of all Stage I–III cases)—OR 1.25 (1.21–1.29), *p*‐value < 0.0001. Figure 2. Illustrates the differences in YO‐PBA and AO‐PBA with respect to the distribution of (a) race/ethnicity, (b) income and (c) stage.

**TABLE 2:** Summary of multivariable logistic regression model for factors associated with YO‐PBA

Characteristics	Comparison	Odds Ratio (95% CI)	*p*‐Value
Disease	Intra Bile vs. Pancreatic	1.92 (1.83–2.02)	<0.0001
	Other Bile vs. Pancreatic	1.23 (1.17–1.29)	<0.0001
Sex	Male vs. Female	1.29 (1.25–1.34)	<0.0001
Race/ethnicity	Non‐Hispanic Black vs. Non‐Hispanic White	1.72 (1.64–1.80)	<0.0001
	Asian vs. Non‐Hispanic White	1.59 (1.47–1.72)	<0.0001
	Hispanic vs. Non‐Hispanic White	2.25 (2.13–2.38)	<0.0001
Community median income	<40K vs. ≥63K	1.08 (1.03–1.13)	0.0025
	40–50K vs. ≥63K	1.11 (1.06–1.16)	<0.0001
	50–63K vs. ≥63K	1.00 (0.95–1.04)	0.8606
Residence area	Metro vs. Urban	0.94 (0.90–0.99)	0.0151
	Rural vs. Urban	0.87 (0.76–0.99)	0.0384
Charleson‐Deyo score	0 vs. ≥1	2.17 (2.08–2.25)	<0.0001
Stage	Stage IV vs. I–III	1.25 (1.21–1.29)	<0.0001

### 3.3 Survival analysis

**FIGURE 3:**
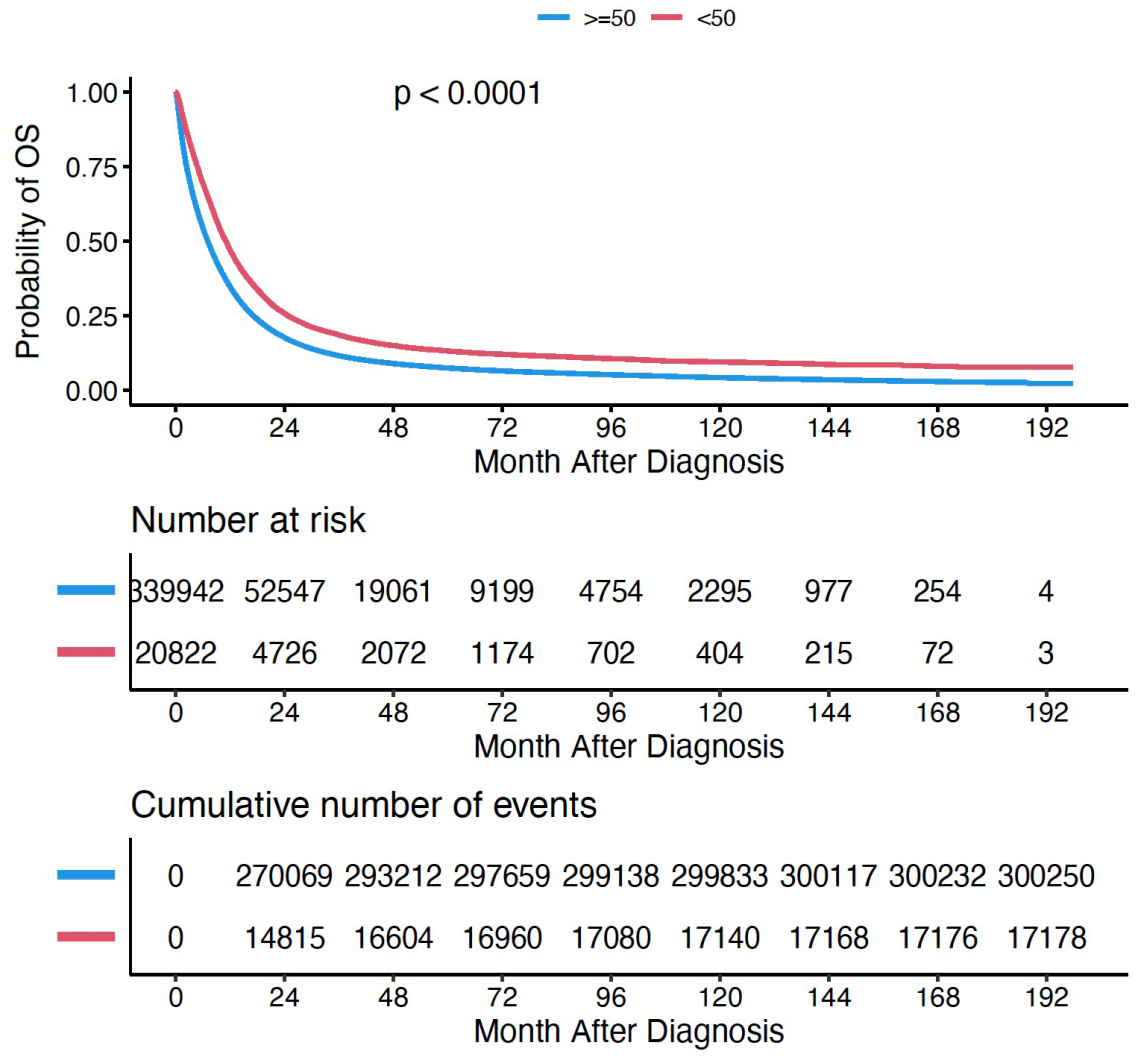
Kaplan–Meier curves comparing survival outcomes for YO‐PBA versus AO‐PBA

Median overall survival (OS) for YO‐PBA vs. AO‐PBA was 11.0 months (95% CI 10.8–11.3) versus 7.06 months (95% CI 7.03–7.13), *p*‐value < 0.0001. Median follow‐up times for YO‐PBA and AO‐PBA were 37.2 months and 28.4 months respectively (range 0.1–197.7 months for both). The Kaplan–Meier curves for YO versus AO‐PBA is shown in Figure 3. Survival was significantly impacted by surgery (median OS 8.2 months without surgery and 31.0 months with surgery in YO‐PBA and 5.3 versus 24.3 months in AO‐PBA).

Regression analysis was performed separately for non‐operable patients and operable patients who underwent curative surgery (selection process outlined in Figure 1). The proportion of operable patients were 15.1% for YO‐PBA and 14.5% AO‐PBA. On univariate analysis, factors associated with overall survival (OS) in non‐operable patients included—sex (male vs. female, HR 1.12, 95% CI 1.08–1.15, *p*‐value < 0.0001), race (non‐Hispanic Black vs. White, HR 1.23, 95% CI 1.06–1.42, *p*‐value = 0.005), income group (lowest vs. highest, HR 1.33, 95% CI 1.27–1.39, *p*‐value < 0.0001), and treatment center type (academic vs. nonacademic center, HR 0.87, 95% CI 0.85–0.90, *p*‐value < 0.0001). Non‐operable status was associated with the highest HR 3.29 (3.16–3.42). Therefore, groups were analyzed separately on the basis of their operability and ability to undergo curative surgery.

On multivariate Cox regression analysis, significant factors associated with survival outcomes for non‐operable YO‐PBA included—sex (male vs. female, HR 1.11, 95% CI 1.06–1.15), income group (lowest vs. highest, HR 1.23, 95% CI 1.13–1.33), treatment center (academic vs. nonacademic center, HR 0.90, 95% CI 0.86–0.94), *p*‐values < 0.0001 for all. These results are described in detail in Table 3. Non‐Hispanic Black race was associated with better survival outcomes versus Non‐Hispanic White race (HR 0.91, 95% CI 0.86–0.97 Black vs. White) in the multivariate model when other factors were adjusted. Other significant factors were insurance status, educational status‐based groups, comorbidity index, and receipt of chemotherapy. As shown Table 3, the HRs associated with sex and socioeconomic status (income, educational status) were higher for YO‐PBA compared to AO‐PBA.

**TABLE 3:** Summary of cox model results for OS for non‐operable patients (*n* = 15,512)

Characteristics	Comparison	YO–PBA HR (95% CI)	*p*‐Value	AO–PBA HR (95% CI)	*p*‐Value
Sex	Male vs. Female	1.11 (1.06–1.15)	<.0001	1.05 (1.04–1.06)	<0.0001
Race/Ethnicity	Non‐Hispanic Black vs. Non‐Hispanic White	0.91 (0.86–0.97)	<.0001	0.97 (0.95–0.98)	0.003
	Asian vs. Non‐Hispanic White	0.90 (0.80–1.01)	<.0001	0.87 (0.85–0.90)	0.07
	Hispanic vs. Non‐Hispanic White	0.74(0.68–0.80)	<.0001	0.82 (0.80–0.83)	<.0001
	Others vs. Non‐Hispanic White	0.87(0.71–1.07)	<.0001	0.89 (0.84–0.94)	0.20
Primary payor	Government vs. private	1.26 (1.20–1.33)	<.0001	1.17 (1.16–1.18)	<0.0001
	Not insured vs. private	1.19 (1.11–1.29)	<.0001	1.09 (1.06–1.12)	<0.0001
Community median income	<40K vs. ≥63K	1.23 (1.13–1.33)	<.0001	1.09 (1.07–1.11)	<0.0001
	40–50K vs. ≥63K	1.05 (0.97–1.12)	<.0001	1.06 (1.05–1.08)	0.23
	50–63K vs. ≥63K	1.03 (0.97–1.10)	<.0001	1.03 (1.02–1.04)	0.34
Percent No high school degree quartiles 2012–2016	≥17.6% vs. <6.3%	1.07 (0.98–1.17)	0.0118	0.98 (0.96–1.00)	0.14
	10.9%–17.5% vs. <6.3%	1.17 (1.08–1.26)	0.84	1.00 (0.99–1.02)	<0.0001
	6.3%–10.8% vs. <6.3%	1.11 (1.04–1.19)	0.02	1.02 (1.00–1.03)	0.002
Residence area	Metro vs. Urban	1.00 (0.94–1.07)	0.73	1.00 (0.99–1.02)	0.90
	Rural vs. Urban	1.00 (0.84–1.18)	0.75	1.01 (0.97–1.04)	0.95
Charleson‐Deyo score	0 vs. ≥1	0.88 (0.84–0.93)	<.0001	0.85 (0.84–0.86)	<0.0001
Stage	Stage I–III vs. Stage IV	0.58 (0.55–0.61)	<.0001	0.57 (0.566–0.578)	<0.0001
Cancer center type	Academic/Research Center vs. Non‐Academic Centers	0.90 (0.86–0.94)	<.0001	0.86 (0.85–0.86)	<0.0001
Chemotherapy	Yes vs. No	0.56 (0.53–0.60)	<.0001	0.44 (0.43–0.44)	<0.0001

Similarly, multivariate Cox regression analysis to assess characteristics associated with survival for operable patients was performed and is outlined in Table 4. Survival outcomes for YO‐PBA were significantly impacted by several factors including sex (male vs. female, HR 1.10, 95% CI 1.01–1.20), income group (lowest vs. highest, HR 1.28, 95% CI 1.07–1.52), treatment center (academic vs. nonacademic center, HR 0.90, 95% CI 0.87–1.03), *p*‐values < 0.0001 for all. The detailed results are outlined in Table 4. Race was not a significant factor influencing outcomes for YO‐PBA patients undergoing curative surgery.

**TABLE 4:** Summary of cox model results for OS for Patients that underwent curative surgery (*n* = 52,330)

Characteristics	Comparison	YO–HR (95% CI)	*p*‐Value	AO–HR (95% CI)	*p*‐Value
Sex	Male vs. Female	1.10 (1.01–1.20)	<0.0001	1.10 (1.08–1.13)	<0.0001
Race/Ethnicity	Non‐Hispanic Black vs. Non‐Hispanic White	1.03 (0.90–1.16)	0.67	0.97 (0.93–1.00)	0.0793
	Asian vs. Non‐Hispanic White	1.00 (0.80–1.24)	0.98	0.86 (0.81–0.92)	<0.0001
	Hispanic vs. Non‐Hispanic White	0.86 (0.73–1.02)	0.087	0.88 (0.84–0.93)	<0.0001
	Others vs. Non‐Hispanic White	1.03 (0.69–1.53)	0.90	0.82 (0.72–0.92)	0.0011
Primary payor	Government vs. private	1.35 (1.22–1.51)	<.0001	1.21 (1.18–1.24)	<0.0001
	Not insured vs. private	0.98 (0.81–1.18)	0.81	1.09 (1.01–1.17)	0.02
Community median income	<40K vs. ≥63K	1.28 (1.07–1.52)	0.01	1.12 (1.07–1.17)	<0.0001
	4050K vs. ≥63K	1.15 (1.00–1.34)	0.063	1.09 (1.05–1.13)	<0.0001
	50–63K vs. ≥63K	1.18(1.029–1.352)	0.018	1.04 (1.01–1.07)	0.0121
Percent no high school degree quartiles 2012–2016	≥17.6% vs. <6.3%	0.87 (0.73–1.04)	1.041	1.03 (0.99–1.07)	0.1772
	10.9%–17.5% vs. <6.3%	0.92 (0.79–1.07)	1.07	1.05 (1.01–1.09)	0.01
	6.3%–10.8% vs. <6.3%	0.97 (0.85–1.10)	1.10	1.033 (1.003–1.064)	0.03
Residence Area	Metro vs. Urban	0.90 (0.80–1.03)	0.12	1.00 (0.97–1.03)	0.96
	Rural vs. Urban	0.85 (0.55–1.32)	0.47	0.97 (0.90–1.05)	0.50
Charleson–Deyo score	0 vs. ≥1	0.89 (0.80–0.99)	0.04	0.88 (0.87–0.90)	<0.0001
Cancer center type	Academic/Research Center vs. Non–Academic Centers	0.95 (0.87–1.03)	0.21	0.89 (0.90–0.92)	<0.0001
chemotherapy	Yes vs. No	0.97 (0.86–1.10)	0.63	0.74 (0.72–0.76)	<0.0001

There was notable differences in survival outcomes based on chemotherapy. Among non‐operable patients, the receipt of chemotherapy was associated with improved outcomes in both YO‐PBA (HR 0.56, 95 % CI 0.53–0.60) and AO‐PBA (HR 0.44, 95% CI 0.43–0.44), *p*‐values < 0.0001—the impact being higher for AO‐PA. Interestingly for operable patients, the impact was significant only for AO‐PBA (HR 0.74, 95% CI 0.72–0.76, *p*‐value < 0.0001) and not YO‐PBA (HR 0.97, 95% CI 0.86–1.10).

## 4 DISCUSSION

The study analyzed the characteristics and outcomes for young onset cancer using a cohort of 20,822 patients with pancreatico‐biliary adenocarcinoma in the National Cancer Database (NCDB). The YO‐PBA constituted 6.8% of the entire cohort which is consistent with the literature.^1^, ^2^, ^6^ To the best of our knowledge, this is the largest and most up‐to‐date study on a cohort of patients with YO‐PBA. Although the reported cases of YO‐PBA did increase, it is reassuring to know that unlike other GI cancers such as YO‐CRC, the increase is not as high as for average onset (AO) disease, suggesting that an epidemiologic shift to earlier age at onset may not be happening.^7^ However, the association with demographic characteristics particularly non‐white racial status and socioeconomic status are similar to what has been reported for other young onset GI cancers and warrants further investigation.^7^ The majority of the patients remain non‐operable and the impact of socioeconomic characteristics on survival outcomes remain significant among this vulnerable population. A recent study of 7307 young‐onset pancreatic cancer patients utilizing Surveillance, Epidemiology and End Results (SEER) had similar demographic findings as our study in terms of age and racial distribution, however, did not include average onset disease for comparison.^8^ Other studies that included both young and average onset disease groups were aimed at assessing the treatment utilization characteristics of this population or studying the factors impacting outcomes for patients able to undergo curative surgery.^6^, ^9^

Specific associations between patient characteristics and YO‐PBA were identified in the present study. Although the majority of the patients were non‐Hispanic Whites as in AO cancer, non‐White races and Hispanic ethnicity had a higher proportion of YO‐PBA compared to non‐Hispanic Whites. This is similar to the association noticed for other YO‐GI cancers such as YO‐colorectal cancer.^7^ The risk factors that have been established for pancreatic cancer include obesity, alcohol use, family history of pancreatic cancer, diabetes mellitus, smoking, and pancreatitis with the strongest dose‐dependent association with alcohol use among patients <45 years versus those <60 years.^10^ Therefore, one could hypothesize the increasing incidence of obesity and variability in exposure to known risk factors at least partly explain the pattern observed in YO‐PBA.^8^ Although obesity prevalence has been increasing overall, the increase is higher among non‐Hispanic Black and Hispanic patients compared to non‐Hispanic Whites.^11^ The neighborhood socioeconomic status is also associated with obesity prevalence with higher rates observed in neighborhoods with lower socioeconomic status.^12^, ^13^ Indeed, neighborhood disadvantages and racial disparities impact substance use and outcomes.^14^ While smoking exposure and the association of smoking with lower socioeconomic status is certainly a factor of relevance in cancer epidemiology, the decreasing prevalence of smoking fails to provide an adequate explanation for trends observed in YO‐PBA.^15^, ^16^ Although male sex was associated with a higher likelihood for YO‐PBA, studies have demonstrated a higher rate of increase among young females compared to males (1.93% vs. 0.77%)—likely attributable to differential exposure to risk factors.^17^ Such observed trends and associations need to be studied more comprehensively to establish the relationship robustly. Unfortunately, the National Cancer Database lacks data about BMI or exposures (diet, substance use) and could not be used to study such an association. The findings from our study can be used to further justify the need for healthcare system restructuring and improving the advocacy efforts for risk factor mitigation.

In terms of disease characteristics, the finding that YO‐PBA is more likely to be Stage IV than Stage I–III is concerning as it has direct implications for treatment and survival. Interestingly, other YO‐GI cancers such as YO‐CRC are also known to be associated with an advanced stage at presentation compared to AO disease.^1^, ^7^, ^18^ It is unclear if this is related to the biology of the disease versus a delay in the timely diagnosis of PBA in a young patient compared to an older patient given the influence of a lower risk profile on medical judgment.^6^ Unlike CRC, where lack of screening could partly explain the higher stage of disease at presentation for YO‐PBA patients, such a case doesn’t exist for PBA. In at least one study comparing the genomic and molecular characteristics of young onset pancreatic cancer versus average onset disease, distinct features such as the higher prevalence of K‐RAS wildtype status and enrichment of targetable mutations (ETV6‐NTRK3, TPR‐NTRK1, SCLA5‐NRG1, ATP1B1‐NRG1 fusions, IDH1 R132C mutation, and mismatch repair deficiency) were identified.^19^ Of 138 patients that underwent germline testing, 31.9% had a pathogenic germline variant and 27.5% tested positive for alterations in cancer susceptibility genes.^19^ BRCA1/BRCA2 and PALB2 comprise the majority of pathologic genetic variants (PGV) identified. These findings have therapeutic implications given the activity of platinum therapy for patients with germline alterations involving BRCA1/BRCA2, and the therapeutic benefit of PARP inhibitors in the maintenance setting. This supports the existence of distinct biology based on the age of presentation although this remains to be definitively established. When assessed by the site of disease, biliary adenocarcinomas were more likely to be young‐onset than pancreatic cancers in the current cohort of pancreatico‐biliary adenocarcinomas. The potential differences in disease biology and higher risk of advanced disease may explain the findings in some studies where YO pancreatic cancer patients were shown to have worse survival compared to AO patients when relevant covariates are adjusted using propensity matching.^1^, ^18^

In the hope to detect cancer at an early age, efforts are ongoing to develop screening tools for aggressive cancers such as pancreatico‐biliary cancers in noninvasive fashions. Higher representation of individuals with lower socioeconomic status among YO‐PBA leads us to postulate the contribution of environmental factors and provide a rationale to explore the role of non‐genomic factors that are associated with tumorigenesis and treatment response. These include characteristics such as microbiome (tissue‐based or non‐tissue‐based).^20^, ^21^ Various environmental factors (diet, obesity, medications) may alter the gut microbiome, in turn influencing microbial gene expression patterns and the immune microenvironment, and making the host susceptible to carcinogenesis.^22^

As has been shown in other US studies, most YO‐cancer patients had private insurance as opposed to the AO‐group where the majority had Government insurance.^9^ The prevalence of comorbidities was also lower for the YO‐group compared to AO. While these factors (private insurance and lower prevalence of comorbidity) may contribute to better rates of multimodality treatment and survival outcomes, research does show increased treatment rates even after adjusting for these factors.^6^, ^9^ Despite increased access to treatments overall, barriers do exist and the socioeconomic factors (indicated by the income and educational groups in the NCDB) impact the outcomes for these patients, more so than AO patients as indicated by the higher HRs, especially among patients not able to receive curative surgery. These may be related to competing commitments such as family or household responsibilities, the need to take time off from work, financial obligations, and psychological distress associated with the diagnosis.^9^ To what extent these barriers impact survival in comparison to the biology of the disease needs to be determined. Female sex, non‐White race status, and lower socioeconomic status do independently impact the access to treatment and should be recognized as target areas to improve access to treatment for these patients.^9^ Worse outcomes for Black patients on univariate analysis and reversal of this finding on multivariate modeling suggests that socioeconomic status, insurance, access to care and comorbidities may be a stronger driver of health outcomes. Therefore continued efforts in reducing disparities and improving social determinants of health are needed.

It is notable that the use of chemotherapy was less impactful among YO non‐operable patients compared to AO non‐operable patients, and did not appear to impact the survival outcomes for YO‐PBA that underwent surgery. Definitive conclusions can only be drawn after further investigations to validate these findings. However, the results do suggest the crucial role of surgery for YO‐PBA, and support intensifying research efforts to detect the cancer at an earlier (operable) stage.

Historically, a limitation of studies in the YO‐PBA space has been the variability in defining the young onset group. There is also heterogeneity in the patient cohorts, treatments received, and variability in reporting of risk factors for cancers. Of late, the age cut‐off of 50 years has been used more or less consistently to define YO‐PBA and was used for the present study as well.^19^ The limitations of the present study pertain to the retrospective nature of the data and the possibility of unmeasured confounders. However, the NCDB offers a large volume of patients to study the epidemiological factors associated with the disease occurrence and outcomes. In terms of survival and follow‐up, NCDB tries to attain 90% adherence to mortality reporting consistent with Commission on Cancer (CoC) quality standards and thus likely reflects an acceptable estimate of real‐world outcomes. The use of multiple surrogates for socioeconomic status (income, education, geographical location) and their respective adjustments during analysis improved the robustness and generalizability of our results. The NCDB doesn’t include any exposure data (substance use, diet) or results for molecular testing which limits our ability to look for associations and potential causal factors for young‐onset disease. Molecular analyses focusing on this subset to identify actionable aberrations and differentiate them from adult‐onset are important future directions.

In summary, in a large national cohort study, we found that cases of YO‐PBA reported in NCDB have increased over the years. Socioeconomic factors significantly impact incidence and outcomes for Young‐onset pancreaticobiliary adenocarcinoma (YO‐PBA). The results also suggest that the YO‐PBA may be more chemorefractory than AO‐PBA. Further work is needed to help understand the mechanisms involved and address the disparities to improve the care of patients with YO‐PBA.

## AUTHOR CONTRIBUTIONS

**Thejus Jayakrishnan:** Conceptualization (equal); data curation (equal); formal analysis (equal); methodology (equal); project administration (equal); visualization (equal); writing – original draft (equal); writing – review and editing (equal). **Kanika G. Nair:** Conceptualization (equal); methodology (equal); writing – original draft (equal); writing – review and editing (equal). **Suneel D. Kamath:** Conceptualization (equal); methodology (equal); writing – original draft (equal); writing – review and editing (equal). **Wei Wei:** Formal analysis (equal); methodology (equal); validation (equal); visualization (equal); writing – original draft (equal); writing – review and editing (equal). **Bassam N. Estfan:** Writing – original draft (equal); writing – review and editing (equal). **Smitha S. Krishnamurthi:** Writing – original draft (equal); writing – review and editing (equal). **Alok A. Khorana:** Conceptualization (equal); funding acquisition (equal); investigation (equal); methodology (equal); project administration (equal); resources (equal); supervision (equal); writing – original draft (equal); writing – review and editing (equal).

## FUNDING INFORMATION

Support for this study was provided by the Sondra and Stephen Hardis Chair in Oncology Research (Alok A. Khorana)

## CONFLICT OF INTEREST

Thejus Jayakrishnan—No disclosures. Kanika G. Nair—No disclosures. Suneel Kamath—consulting or advisory role: Exelixis, Guardant Health. Tempus. Wei Wei––No disclosures. Bassam Estfan—No disclosures. Smitha S. Krishnamurthi—Consulting or Advisory Role––Array BioPharma, Research Funding––Abbvie (Inst); Bristol‐Myers Squibb; Celgene (Inst). Alok A. Khorana—has been paid honoraria directly by Janssen, Halozyme, Pfizer, Bayer, Nektar, and Medscape, currently or during the past 2 years. Dr. Khorana has been paid for any consulting or advisory role by Janssen, Halozyme, Bayer, Pfizer, Pharmacyte Biotech, Pharmacyclics, and Seattle Genetics.

## ETHICS APPROVAL

This study was exempt from institutional review board oversight and the ethical approval process.

## DISCLAIMER

The American College of Surgeons and the Commission on Cancer have not verified and are not responsible for the analytic or statistical methodology employed, or the conclusions drawn from these data by the investigator.

## PRIOR PRESENTATION

The study was presented at the poster discussion session as part of the American Society of Clinical Oncology Annual Meeting, 2022, and received the ASCO Conquer Cancer Award

## Data Availability

Authors Thejus Jayakrishnan and Alok A. Khorana had full access to all the data in the study. We take full responsibility for the integrity of the data. The data that support the findings of this study are available from the corresponding author upon reasonable request.

## References

[1] Ansari D , Althini C , Ohlsson H , Andersson R . Early‐onset pancreatic cancer: a population‐based study using the SEER registry. Langenbecks Arch Surg. 2019;404(5):565‐571. doi:10.1007/s00423-019-01810-0 31377855 PMC6713682

[2] Cancer of the Pancreas ‐ Cancer Stat Facts. SEER. Accessed September 20, 2021. https://seer.cancer.gov/statfacts/html/pancreas.html

[3] Cancer of the Liver and Intrahepatic Bile Duct ‐ Cancer Stat Facts. SEER. Accessed May 7, 2022. https://seer.cancer.gov/statfacts/html/livibd.html

[4] Deyo RA , Cherkin DC , Ciol MA . Adapting a clinical comorbidity index for use with ICD‐9‐CM administrative databases. J Clin Epidemiol. 1992;45(6):613‐619. 1607900 10.1016/0895-4356(92)90133-8

[5] Earlier Ovarian Cancer Diagnoses and Treatment Seen After ACA Implementation. ASCO. Published May 31, 2019. Accessed June 29, 2020. https://www.asco.org/about‐asco/press‐center/news‐releases/earlier‐ovarian‐cancer‐diagnoses‐and‐treatment‐seen‐after‐aca

[6] Ordonez JE , Hester CA , Zhu H , et al. Clinicopathologic features and outcomes of early‐onset pancreatic adenocarcinoma in the United States. Ann Surg Oncol. 2020;27(6):1997‐2006. doi:10.1245/s10434-019-08096-y 31894482

[7] Kamath SD , Torrejon N , Wei W , et al. Racial disparities negatively impact outcomes in early‐onset colorectal cancer independent of socioeconomic status. Cancer Med. 2021;10(21):7542‐7550. doi:10.1002/cam4.4276 34647438 PMC8559495

[8] LaPelusa M , Shen C , Arhin ND , et al. Trends in the incidence and treatment of early‐onset pancreatic cancer. Cancers (Basel). 2022;14(2):283. doi:10.3390/cancers14020283 35053447 PMC8773833

[9] Saadat LV , Chou JF , Gonen M , et al. Treatment patterns and survival in patients with early‐onset pancreatic cancer. Cancer. 2021;127(19):3566‐3578. doi:10.1002/cncr.33664 34228820 PMC8711090

[10] McWilliams RR , Maisonneuve P , Bamlet WR , et al. Risk factors for early‐onset and very‐early‐onset pancreatic adenocarcinoma: a pancreatic cancer case‐control consortium (PanC4) analysis. Pancreas. 2016;45(2):311‐316. doi:10.1097/MPA.0000000000000392 26646264 PMC4710562

[11] Freedman DS , Khan LK , Serdula MK , Ogden CL , Dietz WH . Racial and ethnic differences in secular trends for childhood BMI, weight, and height. Obesity (Silver Spring). 2006;14(2):301‐308. doi:10.1038/oby.2006.39 16571857

[12] Mohammed SH , Habtewold TD , Birhanu MM , et al. Neighbourhood socioeconomic status and overweight/obesity: a systematic review and meta‐analysis of epidemiological studies. BMJ Open. 2019;9(11):e028238. doi:10.1136/bmjopen-2018-028238 PMC6886990 31727643

[13] Akil L , Ahmad HA . Effects of socioeconomic factors on obesity rates in four southern states and Colorado. Ethn Dis. 2011;21(1):58‐62. 21462731 PMC3101796

[14] Collins SE . Associations between socioeconomic factors and alcohol outcomes. Alcohol Res. 2016;38(1):83‐94. 27159815 10.35946/arcr.v38.1.11 PMC4872618

[15] CDC . Current cigarette smoking among adults in the United States. Centers for Disease Control and Prevention. Published March 16, 2022. Accessed May 30, 2022. https://www.cdc.gov/tobacco/data_statistics/fact_sheets/adult_data/cig_smoking/index.htm

[16] Cornelius ME , Loretan CG , Wang TW , Jamal A , Homa DM . Tobacco Product use among adults ‐ United States, 2020. MMWR Morb Mortal Wkly Rep. 2022;71:397‐405. doi:10.15585/mmwr.mm7111a1 35298455 PMC8942309

[17] Gaddam S , Abboud Y , Oh J , et al. Incidence of pancreatic cancer by age and sex in the US, 2000‐2018. JAMA. 2021;326(20):2075‐2077. doi:10.1001/jama.2021.18859 34689206 PMC8543346

[18] Eguchi H , Kobayashi S , Gotoh K , Noda T , Doki Y . Characteristics of early‐onset pancreatic cancer and its association with familial pancreatic cancer and hereditary pancreatic cancer syndromes. Ann Gastroenterol Surg. 2020;4(3):229‐233. doi:10.1002/ags3.12326 32490337 PMC7240141

[19] Varghese AM , Singh I , Singh R , et al. Early‐onset pancreas cancer: clinical descriptors, genomics, and outcomes. J Natl Cancer Inst. 2021;113(9):1194‐1202. doi:10.1093/jnci/djab038 33755158 PMC8418394

[20] Wheatley RC , Kilgour E , Jacobs T , et al. Potential influence of the microbiome environment in patients with biliary tract cancer and implications for therapy. Br J Cancer. 2022;126(5):693‐705. doi:10.1038/s41416-021-01583-8 34663949 PMC8888758

[21] Guo W , Zhang Y , Guo S , et al. Tumor microbiome contributes to an aggressive phenotype in the basal‐like subtype of pancreatic cancer. Commun Biol. 2021;4(1):1‐13. doi:10.1038/s42003-021-02557-5 34465850 PMC8408135

[22] REACCT Collaborative , Zaborowski AM , Abdile A , Adamina M , et al. Characteristics of early‐onset vs late‐onset colorectal cancer: a review. JAMA Surg. 2021;156(9):865‐874. doi:10.1001/jamasurg.2021.2380 34190968

