# Correction to “Comparison of characteristics and outcomes of young‐onset versus average onset pancreatico‐biliary adenocarcinoma”

**DOI:** 10.1002/cam4.6193

**Published:** 2023-07-05

Jayakrishnan T, Nair KG, Kamath SD, et al. Comparison of characteristics and outcomes of young‐onset versus average onset pancreatico‐biliary adenocarcinoma. Cancer Med 2023;12:7327–7338. doi: 10.1002/cam4.5418

The funding information has been revised to read:

Sondra and Stephen Hardis Chair in Oncology Research and Porter Family Fund.

The authors apologize for this error.

